# Rhodamine B Dye-Functionalized Hydrophobic Carbon Quantum Dots with Dual Emission for White-Light Organic Optoelectronic Devices

**DOI:** 10.3390/nano16080482

**Published:** 2026-04-18

**Authors:** Walaa Al-Masri, Alaa Y. Mahmoud

**Affiliations:** Department of Physical Sciences, College of Science, University of Jeddah, Jeddah 21589-80327, Saudi Arabia; walmasri@uj.edu.sa

**Keywords:** hydrophobic carbon quantum dots, rhodamine B composites, photoluminescence, dual-emission, white-light emission, surface functionalization, quantum yield, solvothermal synthesis, organic optoelectronic materials

## Abstract

Hydrophobic carbon quantum dots (hbCQDs) with tunable photoluminescence were synthesized via a solvothermal approach and further hybridized with Rhodamine B (RhB) to extend emission into the visible range. The hbCQDs exhibit quasi-spherical morphology with an average particle size of 8 nm and predominantly disordered graphitic structure, as confirmed by TEM and XRD analyses. FTIR and XPS characterizations reveal surface functional groups including C–N, C=O/C–O, and S–H, which govern the photoluminescence properties. Pure hbCQDs display blue emission at 453 nm under excitation, with a quantum yield (QY) of 6.2%. Incorporation of RhB leads to dual-emission behavior: the surface-state emission remains in the blue region, while molecular-state emission from RhB appears in the orange-red region. The 0.2 mL RhB–CQD composite exhibits optimal properties, including a QY of 13% and a production yield of 82%, emitting white light under 365 nm UV excitation. Increasing RhB loading to 0.4 mL results in a shift in emission peaks and a reduced QY (<9%), with weaker orange fluorescence. These findings demonstrate that controlled RhB hybridization effectively tunes the emission spectrum of hbCQDs, offering a simple and reproducible strategy to achieve dual-color and white-light emission. The optimized hbCQDs/RhB composites hold significant potential for applications in hydrophobic media-compatible organic optoelectronics, light-emitting devices, and bioimaging.

## 1. Introduction

Carbon quantum dots (CQDs) have emerged as an important class of zero-dimensional carbon-based nanomaterials due to their tunable photoluminescence, excellent chemical stability, and low toxicity [1,2,3]. Their distinctive optical characteristics, including size-dependent emission and surface-state-mediated fluorescence, have enabled a wide range of applications in bioimaging, chemical sensing, photocatalysis, and optoelectronic devices [4,5,6,7]. However, most reported CQDs are inherently hydrophilic [8,9,10], which limits their compatibility with nonpolar environments and hinders their integration into hydrophobic matrices and organic optoelectronic systems. In contrast, hydrophobic CQDs offer distinct advantages in such applications, as their compatibility with nonpolar media facilitates improved dispersion, interfacial interaction, and device stability. Despite these advantages, extending the emission of CQDs toward the low-energy visible and red spectral regions remains a significant challenge. Typically, pristine CQDs exhibit dominant blue emission arising from surface defect states [11,12], whereas red or white-light emission is relatively rare and often requires complex surface passivation [13,14,15] or hybridization strategies [16,17,18].

To overcome these limitations, hybrid nanostructures that combine CQDs with luminescent organic dyes have recently attracted considerable attention. In such systems, CQDs can function as broadband surface-state emitters or energy donors, whereas organic dyes contribute strong and well-defined molecular fluorescence in the visible region. Among the available dyes, Rhodamine B (RhB) has emerged as a promising candidate due to its high molar absorption coefficient, excellent photostability, and intense emission in the orange–red region of the spectrum [19,20,21].

The spectral overlap between the emission of CQDs and the absorption band of RhB facilitates efficient interfacial photophysical processes such as Förster resonance energy transfer (FRET) or photoinduced charge transfer, which can broaden the overall emission bandwidth and improve light-harvesting efficiency [22,23,24]. Through these interactions, the excited-state energy generated within CQDs can be partially transferred to RhB molecules, thereby modifying exciton recombination pathways and extending the emission toward longer wavelengths. As a result, CQD–RhB hybrid systems can exhibit dual-emission behavior and broadened photoluminescence spectra, which are highly desirable for achieving tunable visible-light emission and potentially near white-light generation under ultraviolet excitation [11,12].

Although organic dyes such as RhB provide strong visible-light emission [25,26], the direct incorporation of dye molecules into CQD systems to form stable and reproducible composites without fluorescence quenching remains insufficiently explored. In particular, systematic investigations on how the concentration of RhB influences the photophysical properties, quantum yield, and emission tunability of hydrophobic CQDs are still limited. Most reported CQD–RhB hybrid systems are based on hydrophilic CQDs and rely on post-synthetic dye incorporation, which often leads to aggregation-induced fluorescence quenching and limited control over emission behavior. In contrast, the present work employs hydrophobic CQDs and an in situ incorporation of RhB during solvothermal synthesis, enabling improved dye dispersion, reduced quenching at optimal loading, and more stable dual-emission characteristics. Furthermore, the adopted synthesis approach demonstrates good reproducibility, yielding consistent optical properties across multiple batches. This strategy provides a distinct pathway for tuning photoluminescence in nonpolar environments and expands the applicability of CQD-based hybrid systems in organic optoelectronic applications.

In this work, these challenges are addressed by synthesizing hydrophobic carbon quantum dots (hbCQDs) and fabricating hbCQDs/RhB hybrid composites with controlled dye loading. The study aims to elucidate the structural and optical interactions between CQDs and RhB, evaluate the contribution of molecular-state emission to surface-state fluorescence, and identify optimized compositions that enhance quantum yield and broaden visible-light emission. These findings provide valuable insights for designing CQD-based hybrid luminescent materials and advance their potential applications in white-light organic optoelectronic devices and hydrophobic media-compatible photonic systems.

## 2. Experimental Section

### 2.1. Materials

Melamine (MA) (C_3_N_3_(NH_2_)_3)_, dithiosalicylic acid (C_14_H_10_O_4_S_2_), and Rhodamine B (RhB) (C_28_H_31_ClN_2_O_3_) were procured from Shanghai Adamas Reagent Co., Ltd. (Shanghai, China) Acetic acid (CH_3_COOH) was supplied by Guangdong Guanghua Sci-Tech Co., Ltd. (Shantou, China). All reagents were of analytical grade and used as received without further purification. Deionized water was produced using a Milli-Q water purification system (Millipore, Darmstadt, Germany) and was employed consistently throughout all experimental procedures.

### 2.2. Synthesis of Hydrophobic Powder CQDs

**Figure 1:**
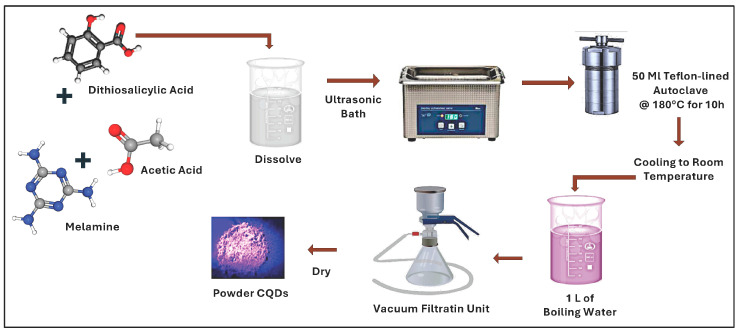
Schematic diagram for the synthesis of carbon quantum dots by Solvothermal method.

The hydrophobic carbon quantum dots (hbCQDs) were synthesized via a solvothermal route [27,28] following the procedure schematically illustrated in Figure 1. Initially, melamine (MA, 0.00079 mol) and thiosalicylic acid (0.0064 mol) were dissolved in 20 mL of acetic acid under ultrasonic irradiation until a homogeneous and transparent solution was obtained, ensuring complete precursor dispersion and molecular-level mixing. Subsequently, the resulting solution was transferred into a 50 mL Teflon-lined autoclave reactor and heated in an oven at 180 °C for 10 h to promote carbonization and nucleation of CQDs under autogenous pressure. After completion of the reaction and natural cooling to room temperature, the as-prepared CQD solution was rapidly injected into 1 L of boiling deionized water (100 °C). This step induced flocculation of the hbCQDs, facilitating the removal of unreacted precursors and residual solvent through phase separation. Aggregated CQDs were thereby formed and isolated. The precipitated CQDs were collected by vacuum filtration and thoroughly washed. Finally, the purified CQD powder was dried in an oven at 70 °C until constant weight was achieved, confirming the effectiveness and reproducibility of the water-washing purification protocol.

### 2.3. Synthesis of Powder CQDs/Rhodamine B Hybrid Composite

To extend the emission profile of the CQDs toward the low-energy (red) region—an area of considerable interest due to its limited availability and typically low production yield—a CQDs/RhB hybrid system was developed. The hybrid materials were synthesized following the same solvothermal protocol applied for the preparation of hydrophobic CQDs, with the incorporation of Rhodamine B (RhB) as a luminescent modifier. Specifically, controlled volumes of RhB (0.2 and 0.4 mL) were introduced into the precursor solution prior to the solvothermal treatment. RhB, initially a solid, was dissolved in deionized water to prepare a 1 mg/mL stock solution, from which 0.2 mL (0.20 mg) and 0.4 mL (0.40 mg) were added to the precursor solution, ensuring precise and reproducible dosing. Although the solvothermal process involves 180 °C for 10 h, a fraction of RhB molecules is preserved and preferentially localizes on the CQD surface, as evidenced by the characteristic orange-red emission in the PL spectra, indicating that the dye retains its luminescent properties without significant degradation. Two hybrid samples were thus fabricated with different RhB loadings to systematically investigate the influence of dye concentration on the optical properties and emission tunability of the resulting CQDs/RhB nanocomposites.

## 3. Instrumentation

X-ray diffraction (XRD) analysis was conducted using a Rigaku Ultima IV diffractometer. High-resolution transmission electron microscopy (HR-TEM) characterization was performed on a JEOL ARM-200F (Japan Electron Optics Laboratory Co., Ltd., Tokyo, Japan) operating at 200 kV and equipped with dual Cs aberration correctors. Micrographs were captured using an Orius camera (Gatan, Inc., Pleasanton, CA, USA), and subsequent image analysis and data processing were carried out with Gatan software (version 3.x). Fourier-transform infrared (FTIR) spectra were acquired using a PerkinElmer FTIR spectrometer (PerkinElmer, Inc., an American corporation, Waltham, MA, USA). Samples were mounted on a thin-film holder with the film surface oriented toward the incident IR beam, and spectra were recorded across the 400–4000 cm^−1^ wavenumber range. X-ray photoelectron spectroscopy (XPS) measurements were performed using an XPS system, and spectral deconvolution and analysis were completed with CasaXPS. For XPS characterization, CQD dispersions were drop-cast onto 10 mm × 10 mm indium tin oxide (ITO) substrates and oven-dried prior to measurement. Optical absorption spectra were obtained using a Jasco V-770 UV–Vis–NIR spectrophotometer (JASCO Corporation, Tokyo, Japan). Photoluminescence (PL) measurements were conducted with a Jasco fluorescence spectrometer. The quantum yield (QY) of the CQD samples was determined using a 100 mm integrating sphere coupled to a Jasco ILF-835 spectrofluorometer. All UV–Vis and PL measurements were conducted on solid-state (powder) samples under identical conditions to ensure consistency and comparability. The quantum yield (QY) of the CQD samples was determined using a 100 mm integrating sphere coupled to a Jasco ILF-835 spectrofluorometer, with an excitation wavelength of 365 nm. The inner surface of the integrating sphere was coated with Spectralon^®^, ensuring uniform light reflection and accurate photon collection.

## 4. Results and Discussion

### 4.1. Structural Characterization

#### 4.1.1. X-Ray Diffraction Analysis (XRD)

**Figure 2:**
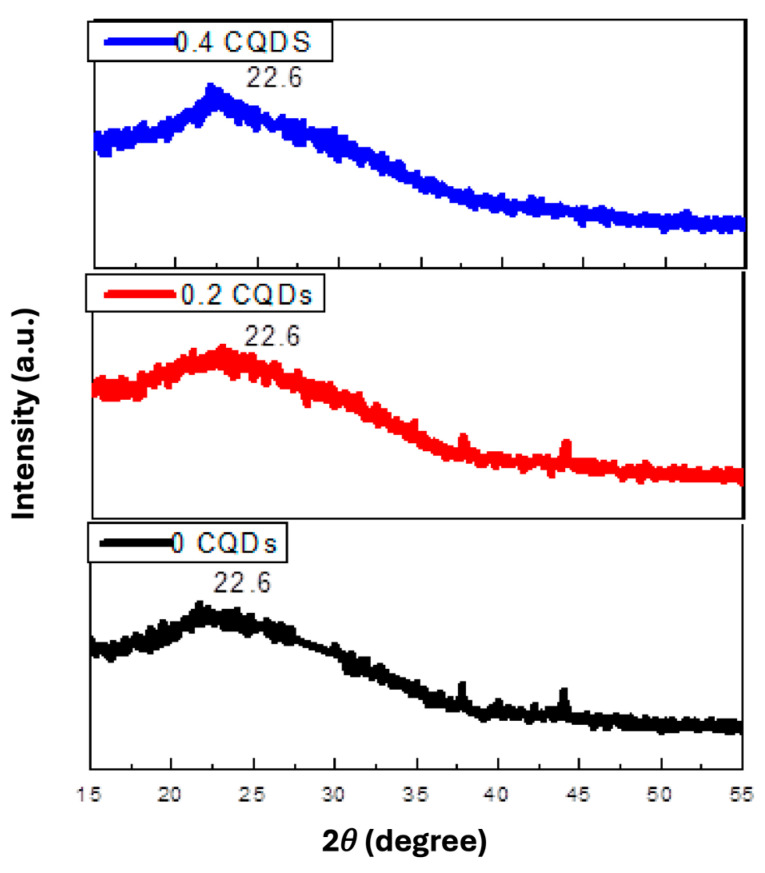
X-ray diffraction (XRD) patterns of hydrophobic carbon quantum dots (hbCQDs) and hbCQDs/Rhodamine B composite solid powders prepared with varying RhB loadings.

The XRD patterns of pure hbCQDs and hbCQDs/RhB composites are presented in Figure 2. The diffraction profile of pristine hbCQDs exhibits a broad peak centered at approximately 22.65°, corresponding to an interlayer spacing (d-spacing) of 0.39 nm, which is indexed to the (002) plane of graphitic carbon. This broadened feature indicates a low degree of crystallinity and the presence of turbostratic or amorphous carbon domains. Additionally, a weak diffraction peak is observed around 41°, with a calculated interlayer spacing of 0.21 nm, attributed to the (100) in-plane lattice reflection. These structural features collectively confirm the formation of carbon quantum dots with predominantly disordered graphitic structures. For the hbCQDs/Rhodamine B composites, the characteristic (002) and (100) diffraction peaks are retained at low RhB loading, indicating that the fundamental carbon framework remains largely unaffected. However, at higher RhB content, the (100) reflection becomes indistinguishable, suggesting increased structural disorder or partial surface coverage by RhB molecules, which may disrupt the in-plane graphitic ordering of the CQDs.

#### 4.1.2. Transmission Electron Microscopy Analysis (TEM)

**Figure 3:**
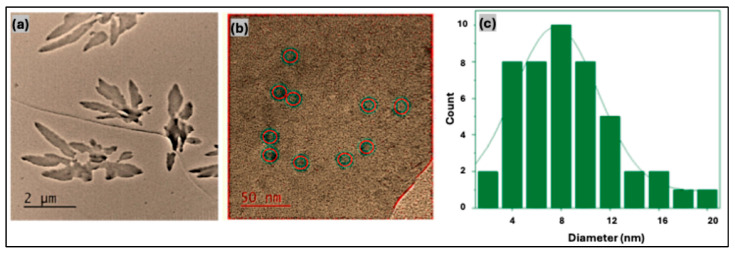
TEM images of hbCQDs: (**a**) low-magnification image (scale bar: 2 μm) showing particle aggregation behavior; (**b**) high-magnification image (scale bar: 50 nm) illustrating quasi-spherical morphology and structural features; red insets showing the particle size distribution and (**c**) particle size distribution histogram obtained from TEM analysis using Gatan software (version 3.x).

Transmission electron microscopy (TEM) was employed to examine the morphology, dispersion behavior, and particle size distribution of the carbon quantum dots. The low-magnification TEM image (scale bar: 2 μm) in Figure 3a illustrates the aggregation behavior of hbCQDs in aqueous media. The particles exhibit a pronounced tendency to form irregular, randomly shaped clusters, reflecting their hydrophobic surface characteristics and limited colloidal stability in water. High-magnification TEM images (Figure 3b) reveal that the hbCQDs are quasi-spherical and partially crystalline in nature. Lattice fringes observed in certain regions indicate localized graphitic ordering within the carbon core. Statistical analysis of particle diameters, derived from multiple TEM micrographs, shows a size distribution ranging from 2 to 20 nm, with an average particle size of approximately 8 nm, as summarized in the histogram presented in Figure 3c.

#### 4.1.3. Fourier-Transform Infrared Analysis (FTIR)

**Figure 4:**
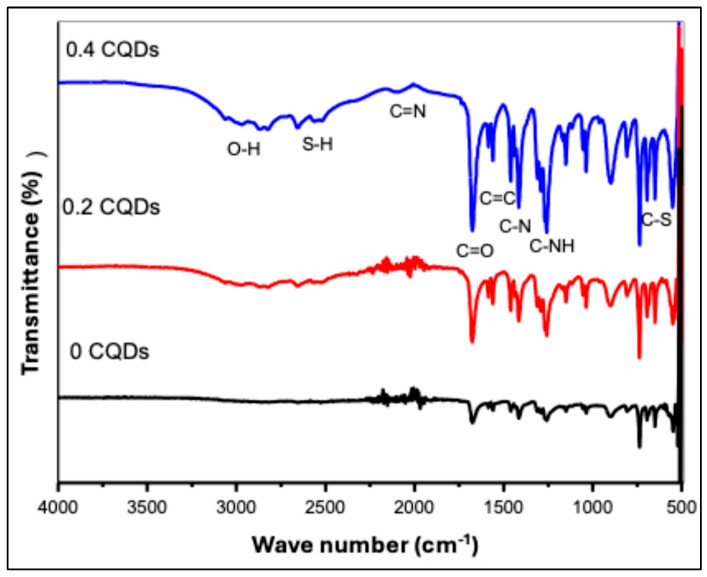
FTIR spectra of hbCQDs and hbCQDs/Rhodamine B composites: pure hbCQDs (black), 0.2 mL RhB–hbCQDs (red), and 0.4 mL RhB–hbCQDs (blue), highlighting the evolution of surface functional groups with increasing RhB content.

Fourier-transform infrared (FTIR) spectroscopy was conducted to identify the surface functional groups of the hydrophobic CQDs. The FTIR spectra of pristine hbCQDs and hbCQDs/RhB composites are presented in Figure 4. The spectrum of pure hbCQDs exhibits relatively weak absorption features, indicating a surface with limited oxygen-containing functionalities. The broad region between 4000 and 2000 cm^−1^ shows minimal prominent peaks, confirming that the structure is predominantly carbonaceous with low surface polarity. This observation is consistent with the hydrophobic nature of the synthesized CQDs [29]. Upon incorporation of a low amount (0.2 mL) of Rhodamine B (RhB), new low-intensity functional group signals emerge. A noticeable O–H stretching vibration appears around 2852 cm^−1^, indicating the introduction of hydrophilic groups associated with RhB molecules. With a further increase in RhB content to 0.4 mL, the O–H stretching band becomes significantly more intense, suggesting enhanced surface functionalization. Consequently, the surface character transitions from hydrophobic to hydrophilic, enabling improved water dispersibility of the composite [30,31]. All samples display characteristic absorption bands corresponding to C≡N stretching (2034 cm^−1^), S–H stretching (2650 cm^−1^), and amide carbonyl (C=O) stretching at 1682 cm^−1^. Additional peaks are observed at 1469 cm^−1^ (C=C), 1415 cm^−1^ (C–N), 1264 cm^−1^ (aromatic C–NH), 1155 cm^−1^ (C–O), and 690 cm^−1^ (C–S). A weak band near 491 cm^−1^ is attributed to S–S vibrations, primarily originating from the dithiosalicylic acid precursor, as confirmed by XPS S 2p spectra, with contributions from RhB considered negligible. Moreover, the asymmetric stretching mode of –N=C=S at 2150 cm^−1^ confirms the presence of isothiocyanate groups derived from RhB [32]. For the composite with higher RhB loading, the spectrum clearly exhibits the characteristic absorption features of RhB in the 1600–1000 cm^−1^ region, reflecting the increased contribution of dye-related functional groups to the overall chemical structure.

#### 4.1.4. X-Ray Photoelectron Spectroscopy Analysis (XPS)

**Figure 5:**
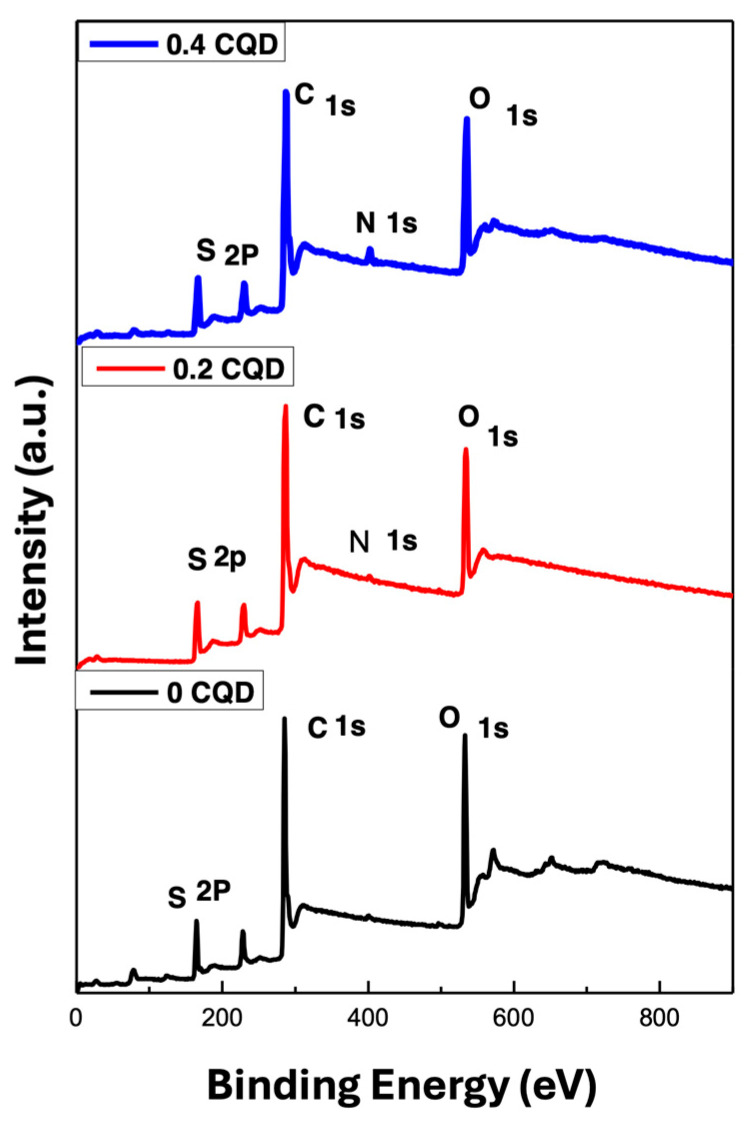
XPS survey spectra of hbCQDs and hbCQDs/Rhodamine B composites: pure hbCQDs (black), 0.2 mL RhB–hbCQDs (red), and 0.4 mL RhB–hbCQDs (blue), illustrating the elemental composition and chemical bonding variations with increasing RhB content.

X-ray photoelectron spectroscopy (XPS) was employed to investigate the elemental composition and chemical bonding of the hbCQDs, as illustrated in Figure 5. The survey spectrum reveals four prominent peaks at binding energies of 531 eV [33], 400 eV [34], 285 eV, and 164 eV [35], corresponding to the O 1s, N 1s, C 1s, and S 2p core levels, respectively. These results confirm the presence of oxygen, nitrogen, carbon, and sulfur in the hbCQDs and provide insight into the surface chemical environment of the synthesized nanomaterials.

**Figure 6:**
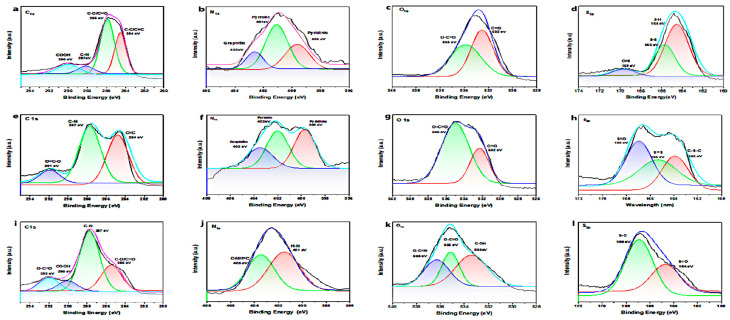
High-resolution XPS spectra of hbCQDs and hbCQDs/RhB composites. (**a**–**d**) Deconvoluted C 1s, N 1s, O 1s, and S 2p spectra of pure hbCQDs showing characteristic functional groups. (**e**–**h**) Spectra of hbCQDs hybridized with 0.2 mL RhB, indicating slight binding energy shifts due to dye interaction. (**i**–**l**) Spectra of hbCQDs with 0.4 mL RhB, showing changes in peak intensity that confirm successful RhB incorporation and surface modification.

The high-resolution XPS spectra of pure hbCQDs were deconvoluted to analyze the chemical states of the constituent elements, as presented in Figure 6. Figure 6a shows the C 1s spectrum of pure hbCQDs, revealing characteristic peaks corresponding to C=C (284.6 eV), C=O/C–O (285.9 eV), and C–N (287.5 eV) bonds. The hbCQDs/RhB composites exhibit similar C 1s profiles; however, a slight positive shift in the C–N binding energy is observed with increasing RhB concentration, suggesting the formation of additional amide linkages within the hybrid structure (Figure 6i).

The O 1s spectra, depicted in Figure 6b, indicate the presence of C=O groups at 532.2 eV in samples with lower RhB content. At higher RhB loading, a new peak emerges at 533.0 eV, corresponding to C–O/C–OH functionalities, reflecting enhanced surface oxidation and functionalization. Figure 6c presents the N 1s spectra, showing contributions from pyrrolic nitrogen (399.6 eV) and pyridinic nitrogen (398.17 eV). With increasing RhB content, the pyridinic nitrogen peak becomes more prominent, indicating the expansion of conjugated aromatic domains within the carbon framework (Figure 6j). The dominance of carbon-related bonding with relatively limited oxygen functionalities supports the low surface polarity and hydrophobic character of the CQDs.

Finally, the S 2p spectra, shown in Figure 6d, confirm the presence of multiple sulfur species, including thiol S–H (164.5 eV), thiophene C–S–C (163.3 eV), disulfide S=S (165.1 eV), and sulfoxide S=O (169.0 eV). With increasing RhB concentration, partial oxidation of sulfur-containing groups occurs, leading to the formation of SOₓ species, which reflects the chemical interaction between CQDs and RhB molecules. This comprehensive XPS analysis demonstrates that RhB incorporation modifies the surface chemistry of hbCQDs, promoting functional group formation and enhanced aromaticity within the composites. These findings indicate that RhB molecules are primarily associated with the CQD surface through a combination of weak chemical interactions (e.g., hydrogen bonding or C–N linkages) and physical adsorption, rather than being incorporated into the carbon core. The retention of characteristic RhB emission further supports the presence of surface-localized molecular states.

### 4.2. Optical Characterization

#### 4.2.1. UV-Vis Absorption Spectroscopy Analysis

**Figure 7:**
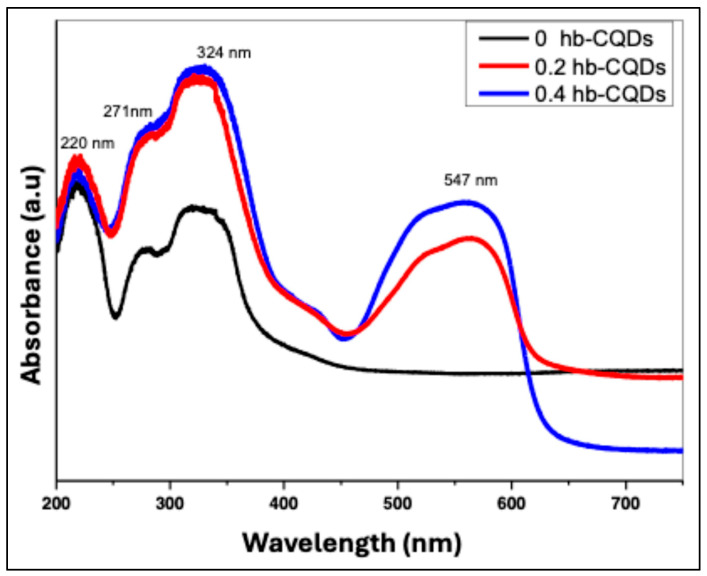
UV–Vis absorption spectra of hbCQDs and hbCQDs/RhB composites: pure hbCQDs (black), 0.2 mL RhB–hbCQDs (red), and 0.4 mL RhB–hbCQDs (blue), highlighting the influence of increasing RhB content on the optical absorption characteristics.

The UV–Vis absorption spectra of the samples can be divided into two distinct regions: a high-energy region spanning 200–400 nm and a low-energy region at wavelengths above 400 nm. Figure 7 presents the absorption profiles of pure hbCQDs and hbCQDs/RhB composites. In the high-energy region, all three samples exhibit similar spectral features with minimal variation. Three prominent absorption peaks are observed, extending into the visible range. The first two peaks, located at 220 nm and 271 nm, are attributed to π–π* transitions of the C=C bonds within the graphitic carbon core of the hbCQDs [31]. A third peak, appearing around 342 nm, is assigned to n–π* transitions arising from surface states, including C=N/C=O, C–O, and C–S functional groups. These features reflect the electronic structure and surface chemistry of the hbCQDs. In the low-energy region above 400 nm, the hbCQDs/RhB composites display a distinct absorption band at 547 nm, corresponding closely to the characteristic absorption of Rhodamine B [36]. This peak confirms successful incorporation of RhB into the CQD matrix and indicates potential for extending photoluminescence into the visible range.

#### 4.2.2. Photoluminescence (PL) Spectroscopy Analysis

**Pure Hydrophobic CQDs Without RhB**

**Figure 8:**
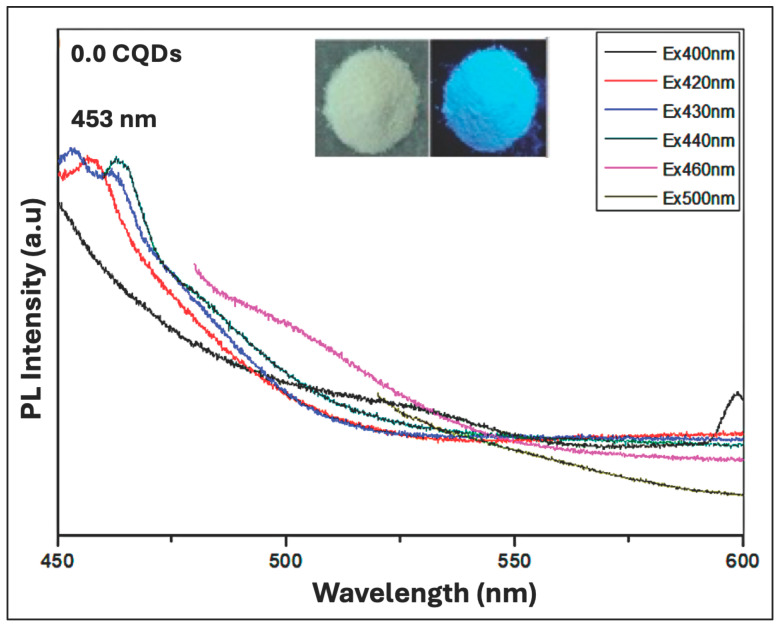
Photoluminescence (PL) spectra of pure hbCQDs (0% RhB). The inset shows comparative photographs of the sample under natural daylight and 365 nm UV illumination, highlighting the characteristic blue emission of the CQDs.

Figure 8 illustrates the PL behavior of pure hbCQDs (0% RhB) under varying excitation wavelengths. Across this excitation range, the CQDs exhibit a characteristic blue emission, with the maximum emission peak centered at approximately 453 nm. The inset displays photographs of the powdered sample under daylight and upon 365 nm UV illumination, clearly demonstrating a vivid blue emission, which confirms the primary luminescent behavior of the CQDs. This emission originates predominantly from surface functional group states rather than the carbon core, with a measured QY of 6.2%. Although dual emission (blue in solution and red in the aggregated state) has been reported for CQDs derived from similar precursors, such behavior strongly depends on synthesis conditions, surface functionalization, and interparticle interactions. In the present case, the hbCQDs exhibit predominantly blue emission, suggesting that aggregation-induced emissive states are not significantly developed, likely due to the specific surface chemistry and hydrophobic nature of the particles.

**Hydrophobic CQDs Composite with RhB of 0.2 mL**

**Figure 9:**
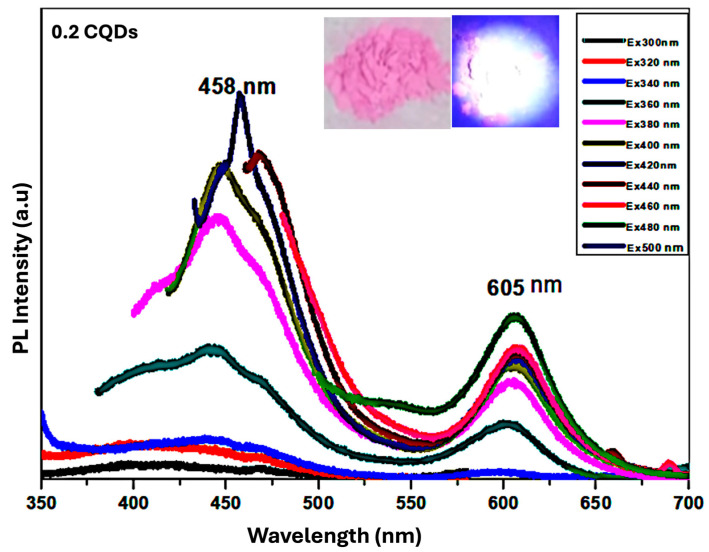
PL spectra of hbCQDs/RhB composite with 0.2 mL RhB. The inset shows comparative photographs of the powder under natural daylight and 365 nm UV illumination, highlighting the color change from bright pink to white emission.

Figure 9 presents the PL spectrum of the hydrophobic CQDs/RhB composite prepared with 0.2 mL RhB. The spectrum exhibits two distinct emission peaks: a higher-energy peak originating from carbon surface states, and a lower-energy peak attributed to molecular states derived from organic fluorophores used as precursors in CQD synthesis. These fluorophores may be covalently bonded to the carbon core or coupled to edge carbon atoms or functional groups, giving rise to n–π* and π–π* transitions. The surface-state emission shows excitation-dependent behavior, with maximum excitation/emission at 458 nm (blue), whereas the molecular-state emission is excitation-independent, with a peak at 605 nm (orange). The inset of Figure 9 shows a photograph of the 0.2 mL RhB–CQD composite powder. Under ambient light, the powder appears bright pink (left), while under 365 nm UV illumination, it emits a strong white light (right), resulting from the combination of the blue and orange emissions from the composite. This composite demonstrates optimal properties, with a QY of 13% and a production yield of 82%, making it a promising candidate for future white-light organic optoelectronic applications.

**Hydrophobic CQDS Composite with RhB of 0.4 mL**

**Figure 10:**
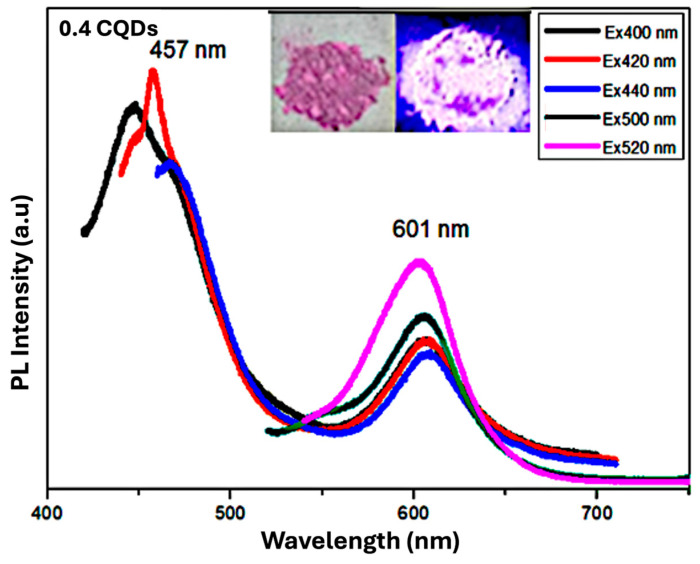
PL spectra of hbCQDs/RhB composite with 0.4 mL RhB. The inset shows comparative photographs of the powder under natural daylight and 365 nm UV illumination, highlighting the color change from purple under ambient light to weak orange emission under UV excitation.

Figure 10 presents the PL spectra of the hydrophobic hbCQDs/RhB composite prepared with 0.4 mL RhB. The spectrum displays two emission peaks: a higher-energy peak arising from carbon surface states and a lower-energy peak associated with molecular states from the incorporated RhB. Compared to the 0.2 mL RhB–hbCQDs sample, the positions of both peaks show slight shifts, reflecting the higher RhB loading. The QY of this sample is reduced to below 9%, indicating decreased emission efficiency at higher dye content. The inset of Figure 10 shows a photograph of the composite powder. Under ambient light (left), the powder appears purple, while under 365 nm UV illumination (right), it emits a weak orange fluorescence. This observation demonstrates that increasing RhB content alters the emission profile and diminishes the overall photoluminescence intensity.

The measured quantum yields were 6.2% for pure hbCQDs, 13% for the 0.2 mL RhB composite, and <9% for the 0.4 mL RhB composite. The observed reduction in quantum yield (<9%) and weaker orange emission for the 0.4 mL RhB-CQD composite are attributed to concentration quenching effects. At higher RhB loadings, dye molecules can aggregate on the CQD surface, leading to nonradiative energy transfer pathways, self-absorption, or inner filter effects. These processes reduce the efficiency of radiative recombination, thereby decreasing the overall emission intensity and quantum yield.

## 5. Conclusions and Future Work

In this study, hydrophobic carbon quantum dots were successfully synthesized via a solvothermal method and subsequently hybridized with Rhodamine B (RhB) to achieve tunable photoluminescence. The pure hbCQDs exhibited blue emission at 453 nm with a quantum yield of 6.2%, while the incorporation of 0.2 mL RhB enabled dual-emission, combining surface-state blue emission and molecular-state orange emission, resulting in white-light emission with a QY of 13% and a production yield of 82%. Higher RhB loading (0.4 mL) caused slight peak shifts and reduced QY (<9%), demonstrating the critical influence of RhB concentration on optical performance. Structural and chemical analyses (TEM, XRD, FTIR, XPS) confirmed the quasi-spherical morphology, partially crystalline carbon core, and the presence of functional groups (C–N, C=O/C–O, S–H) that mediate photoluminescence. Overall, these results indicate that controlled RhB hybridization offers a simple and reproducible strategy to extend the emission of hbCQDs into the visible spectrum, making them suitable candidates for optoelectronic applications, including white-organic-light-emitting devices and hydrophobic media-compatible photonic systems. The confirmed hydrophobic nature of the CQDs further enhances their suitability for applications in nonpolar and organic optoelectronic systems.

Future investigations should focus on optimizing the emission balance between surface-state and molecular-state contributions to maximize white-light efficiency. The stability of hbCQDs/RhB composites under prolonged UV exposure, varying temperatures, and different solvent environments should be systematically evaluated. Furthermore, exploring other organic dyes or co-dopants could expand the emission range and enhance quantum yield. Integration of these composites into device architectures, such as LEDs or flexible optoelectronic systems, will be essential to translate the material’s photophysical properties into practical applications. Additionally, understanding the precise mechanism of energy transfer between CQDs and organic fluorophores at the molecular level would provide insights for designing next-generation hybrid luminescent nanomaterials.

## Data Availability

The authors confirm that the data supporting the findings of this study are available and can be accessed within the article.
